# 

**Published:** 1861

**Authors:** 


### CHICAGO MEDICAL JOURNAL.

**Terms, $2.00 per Annum.**

### CONTENTS OF THE JUNE & JULY NUMBERS.

ORIGINAL COMMUNICATIONS.

PAGE.

An Essay on the Nature and Treatment of Erysipelas. By Jas. S. Whitmire, M. D., of Metamora, Ills. .... 319

Case of Scrofulous Ulcers of the Leg, with Injury of the Ankle Joint—Amputation for. By L. B. Brown, M. D., Iroquois, Ills. .... 328

Removal of four inches of the Tibia, for Necrosis. By M. M. Eaton, M. D., of Peoria, Ills. .... 331

A case of Shoulder Presentation, with Prolapse of Cord. Version by External Manipulation. Treatment by Position. Death of Child. By H. Webster Jones, A. M., M. D. .... 332

Diphtheria. By Dr. J. J. Morgan, of Windham, Iowa .... 333

"Ex Opthalmie Goitre." Reported to the Chicago Academy of Medical Sciences, March, 1861. By R. C. Hamill, M. D. .... 338

"Simila Similibus Curantur," or Like is Cured by Like. Read before the McLean County Medical Society. By C. R. Parks, M. D. .... 382

De Witt County Medical Society .... 348

BOOK NOTICE.

A Treatise on Human Physiology. By John C. Dalton, Jr., M. D., Prof. of Physiology, etc. .... 349

EDITORIAL.

Editorial and Miscellaneous .... 354

SELECTED.

Hemorrhage from Gun-Shot Wound .... 393

Spina Bifida .... 394

On Uræmia. By Prof. Jaksch .... 396

Pus Corpuscles in the Air; An Aeroscopic Study. By Docent Dr. Theoph. Eislet in Prag .... 397

On Atmospheric Corpuscles. By F. M. Pouchet .... 401

Extract from the Sanitary Report to the Surgeon-General. By Surgeon Tripler, M. D., U. S. A. .... 406

The Right Man for the Right Place! .... 409

Use of Cathartics.—Dr. Ware's Lectures .... 413

Sanitary Condition of Troops .... 421

Palliative Treatment of Asthma. By T. L. Pridham, Esq., Bideford, North Devon .... 422

Midwifery and the Diseases of Women, etc. .... 423

Milk-Sickness. Valedictory Address by Isaac Mendenhall, M. D., Ashland, Ind. .... 431

The Cause of Milk-Sickness. By S. C. Chase, M. D., Harmer, Ohio .... 438

On the Treatment of Tetanus .... 440

